# Changes in Coverage and Cost-Related Delays in Care for Latino Individuals After Elimination of the Affordable Care Act’s Individual Mandate

**DOI:** 10.1001/jamanetworkopen.2022.1476

**Published:** 2022-03-08

**Authors:** Alexander N. Ortega, Jie Chen, Dylan H. Roby, Karoline Mortensen, Alexandra C. Rivera-González, Arturo Vargas Bustamante

**Affiliations:** 1 Department of Health Management and Policy, Dornsife School of Public Health, Drexel University, Philadelphia, Pennsylvania; 2 Department of Health Policy and Management, School of Public Health, University of Maryland, College Park; 3 Department of Health, Society, and Behavior, Program in Public Health, University of California, Irvine; 4 Department of Health Management and Policy, Herbert Business School, University of Miami, Florida; 5 Department of Health Policy and Management, Fielding School of Public Health, University of California, Los Angeles

## Abstract

This cross-sectional study examines changes in levels of health care coverage and cost-related delays in care for Latino individuals after elimination of the individual coverage mandate from the Affordable Care Act (ACA).

## Introduction

The Patient Protection and Affordable Care Act (ACA) has been associated with improvements in health insurance coverage and access to care. However, inequities persist.^1^ Studies show that while Latino individuals had significant gains in insurance coverage and access to care, they lag far behind non-Latino Black and White populations.^1,2,3,4^ Since 2010, several changes have occurred in the ACA because of legislative, executive, and court actions. It is important to continue assessing its progress in improving insurance coverage for all US residents and to monitor health care inequities. We analyzed 2019 National Health Interview Survey (NHIS) data^5^ and compared observations with prior periods to examine whether improvements in insurance coverage and access to care continued for Black, Latino, and White populations after the 2019 elimination of the individual mandate.

## Methods

In this cross-sectional study, we grouped 2011-2019 NHIS data by the period before the national ACA implementation (2011-2013), the start of the ACA implementation (2014-2015), the implementation of the health insurance mandate (2016-2018), and the year the individual mandate was eliminated (2019). We limited the sample to participants aged 18 to 64 years. All results were nationally representative. Since NHIS is publicly available with deidentified observations, the Drexel University human research protection program deemed it exempt from institutional review board approval. This study followed the Strengthening the Reporting of Observational Studies in Epidemiology (STROBE) reporting guideline for cross-sectional studies.

We estimated weighted predictive probabilities for the following 4 measures according to self-reported race and ethnicity during the 4 periods: (1) being currently uninsured, (2) having a usual source of care, (3) any emergency department (ED) visit in the past year, and (4) any delay of care due to cost in the past year. Usual source of care is a global measure that does not differentiate types of care. Confidence intervals were used to measure uncertainty. Data analyses were performed using Stata statistical software, version 16.0 (StataCorp LLC).

## Results

Our final sample using NHIS 2011 to 2018 data included 318 056 adults (mean [SD] age, 41.5 [13.1] years). The unweighted sample consisted of 50 104 (15.8%) Black, 64 073 (20.2%) Latino, and 203 879 (64.1%) White individuals; 172 921 (54.4%) were females. Our final sample using NHIS 2019 data included 20 600 adults (mean [SD] age, 43.2 [13.2] years). The unweighted sample for 2019 consisted of 2664 (12.9%) Black, 3516 (17.1%) Latino, and 14 420 (70.0%) White individuals; 10 765 (52.3%) were females.

The percentage of uninsured individuals decreased from the period before the ACA was implemented (19.5% in 2011-2013) until the period when the individual insurance mandate was enforced (12.3% in 2016-2018). However, in 2019, the year the mandate was eliminated, there was a 3–percentage-point increase from the prior period in the probability of being uninsured for everyone (from 12.3% to 15.0%) (Figure). Between the periods of 2016-2018 and 2019, Latino persons had a 5–percentage-point increase in the probability of being uninsured (from 25.0% to 30.1%), and that probability was more than double the probability for Black (14.0%) and White (9.9%) populations in 2019. For ED visits, Black and Latino populations experienced a 3–percentage point and 2–percentage point increase between 2016-2018 and 2019 (Black individuals, from 27.0% to 29.5%; Latino individuals, from 19.0% to 21.4%).

**Figure.**
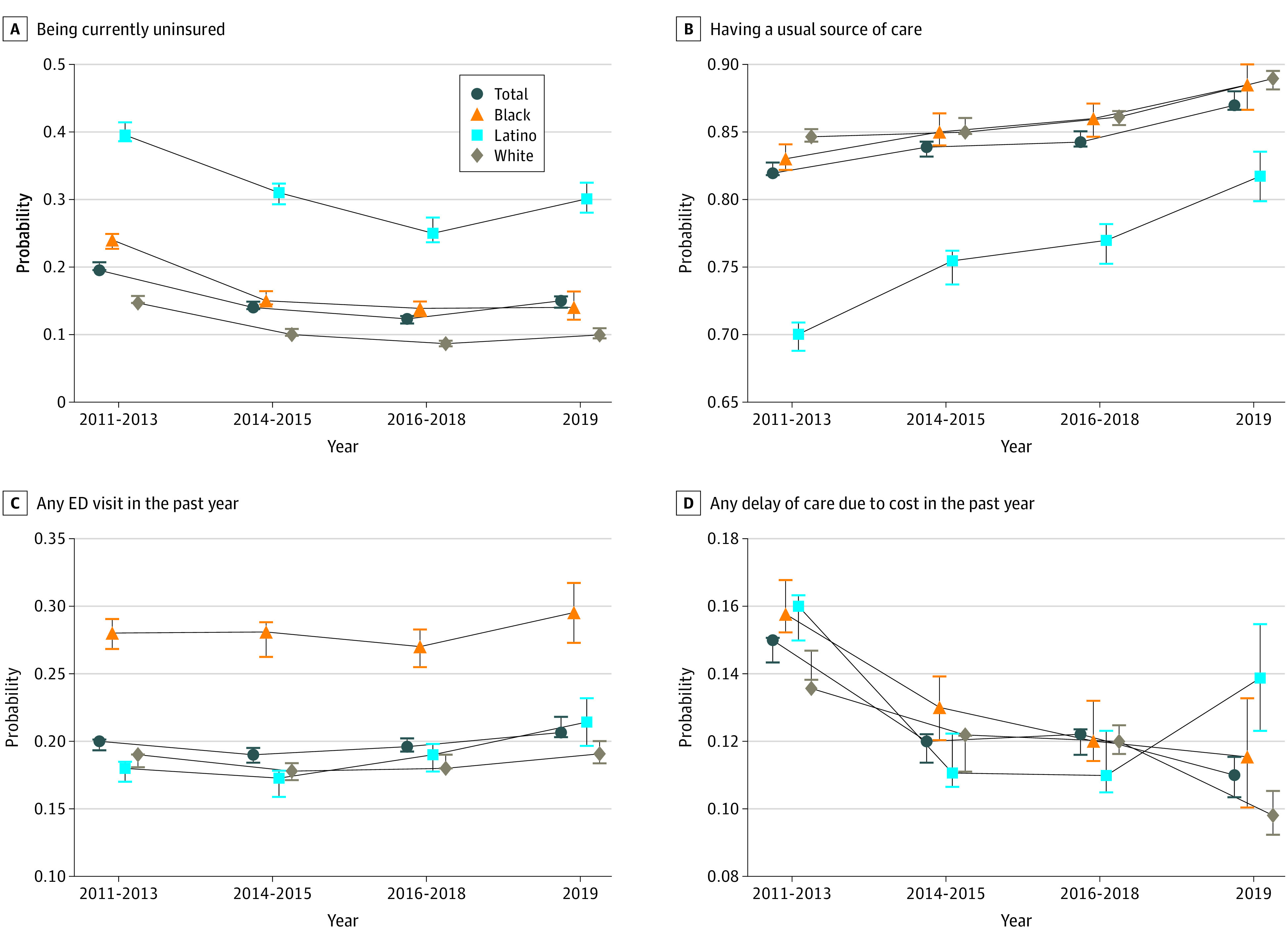
Weighted Probabilities of Self-reported Health Care Access and Utilization by Race and Ethnicity Among Adults Aged 18 to 64 Years Data are from the 2011-2019 National Health Interview Survey (NHIS). All analyses used weighted predictive probabilities. For each data point, 95% CIs are included to show the measure of uncertainty. Results are nationally representative. The Total category includes Black, Latino, and White population groups. The unweighted total number of respondents was 198 514, which consisted of 29 340 Black, 37 670 Latino, and 131 504 White individuals. The NHIS weighting process was updated in the 2019 questionnaire redesign; 2019 NHIS sampling weights were applied to the 2019 period in our analyses. Usual source of care is a global measure that does not differentiate types of care.

Latino populations had a 5–percentage-point increase in the probability of having a usual source of care between 2016-2018 and 2019 (from 77.0% to 81.7%). They also had an increase in the probability of any delay of care due to cost between these periods (from 11.0% to 13.9%); the probability of delay for Black and White populations decreased (Black individuals, from 12.0% to 11.5%; White individuals, from 12.0% to 9.8%).

## Discussion

When we compared observations from the period when the health insurance mandate penalty was in full effect (2016-2018) and the year the mandate was eliminated (2019), we observed that the Latino population had an increase in the probabilities of being uninsured, having an ED visit, and delaying care due to cost, despite an increase in the probability of having a usual source of care. However, usual source of care did not differentiate by types of care. A reversal in these health care equity indicators for Latino populations is evident from these findings.

The elimination of the ACA health insurance mandate may partially explain the increase in the probability of being uninsured for everyone. For Latino populations, the chilling effects of the Trump administration’s public charge regulations and other policies restricting public benefits for immigrants could have played important roles. Policies to reduce out-of-pocket costs, including the continued availability of cost-sharing reductions and enhanced premium tax credits from the 2021-2022 American Rescue Plan, should be continued to address delays in care due to costs.^6^ A limitation of this study is that we did not look at state policy differences. Nevertheless, the findings of this cross-sectional study suggest that encouraging states to expand Medicaid and bolster the health care safety net to improve community-based services will also be beneficial in reversing health care inequities for Latino populations.
